# High CALLY index is independently associated with lower odds of preserved ratio impaired spirometry (PRISm) and provides incremental predictive value: a retrospective observational study

**DOI:** 10.3389/fmed.2026.1897898

**Published:** 2026-07-24

**Authors:** Zelian Zhang, Xiaomin Huang, Wei Tan, Ming Min, Minghua Zhang, Qianfei Liu

**Affiliations:** 1 Department of Pulmonary and Critical Care Medicine, The Central Hospital of Enshi Tujia and Miao Autonomous, Enshi City, Hu Bei, China; 2 Department of Respiratory, The First Affiliated Hospital of Guangxi Medical University, Nanning, Guangxi, China

**Keywords:** biomarker, CALLY index, inverse association, prediction model, preserved ratio impaired spirometry

## Abstract

**Objective:**

To characterize the link of the C-reactive protein-albumin-lymphocyte index (CALLY index) to preserved ratio impaired spirometry (PRISm), and to determine its incremental contribution to PRISm risk prediction in clinical practice.

**Methods:**

A total of 189 participants were included in this study and were classified into the PRISm group (*n* = 70) and the non-PRISm group (*n* = 119) according to the presence or absence of PRISm. Baseline demographic characteristics, comorbidities, and laboratory parameters were collected. Multivariable Logistic regression models with sequential adjustment for confounding factors were used to assess the independent association between the CALLY index and PRISm. Restricted cubic splines (RCS) and subgroup analyses were performed to evaluate the robustness of this association. Receiver operating characteristic (ROC) curve analysis and decision curve analysis (DCA) were used to assess the incremental clinical predictive value of the CALLY index.

**Results:**

The CALLY index was significantly lower in the PRISm group than in the non-PRISm group (1.4 ± 2.5 vs. 4.7 ± 8.1, *P* < 0.001). In the fully adjusted model controlling for 11 confounding factors (adjusted model II), each one-unit increase in the CALLY index as a continuous variable was associated with 16% lower odds of PRISm (OR = 0.84, 95% CI: 0.74–0.95, *P* = 0.007). After dichotomizing the CALLY index, the high-level group showed 73% lower odds of PRISm than the low-level group (OR = 0.27, 95% CI: 0.13–0.57, *P* < 0.001). The RCS curve demonstrated a significant inverse trend between the CALLY index and the odds of PRISm. Subgroup analyses indicated that this inverse association remained robust across populations stratified by age, gender, smoking history, and comorbidities. In addition, after the CALLY index was incorporated into a model containing baseline clinical characteristics, the area under the ROC curve (AUC) increased significantly from 0.611 to 0.703 (*P* = 0.003), and DCA further confirmed a higher net clinical benefit.

**Conclusion:**

A high CALLY index is independently associated with lower odds of PRISm. Integrating the CALLY index into routine clinical assessment can significantly improve the predictive performance for PRISm and provide net clinical benefit.

## 1 Introduction

Amid ongoing efforts to refine the diagnosis and treatment of chronic respiratory diseases, preserved ratio impaired spirometry (PRISm) is drawing considerable investigative attention. Clinically, PRISm denotes a spirometric pattern wherein Forced Expiratory Volume in 1 s (FEV1) drops below 80% of the predicted value despite a preserved post-bronchodilator Forced Expiratory Volume in 1 s/Forced Vital Capacity (FEV1/FVC) ratio ( ≥ 0.70) (1). This distinctive combination of spirometric indicators distinguishes PRISm not only from normal lung function, but also from obstructive ventilatory defects characterized primarily by FEV1/FVC < 0.70, such as Chronic Obstructive Pulmonary Disease (COPD), and from restrictive ventilatory defects mainly defined by reduced total lung capacity. Clinically, PRISm therefore represents a special “transitional state” of pulmonary function as well as a distinct abnormal phenotype. PRISm is commonly observed in the general population, with regional variation in prevalence. The reported global prevalence of PRISm varies from 7.1 to 20.3%; notably, smokers demonstrate a disproportionately higher burden, estimated at around 12.3% (2). PRISm should not be regarded as a “benign state”; prospective data reveal COPD conversion rates of 32.6% among PRISm populations across a 4–5-year follow-up window (3, 4). Data synthesized across several studies confirm that a PRISm diagnosis confers excess mortality burden, encompassing deaths from general causes, respiratory etiologies, and coronary heart disease origins (5–8). Therefore, early identification and intervention for PRISm are of great significance for interrupting the progression of chronic airway disease and reducing the risks of complications and death.

However, technical barriers remain in the early clinical identification and large-scale screening of PRISm. Spirometry, the current diagnostic “gold standard,” has several notable limitations. First, the availability of spirometers in primary healthcare institutions remains insufficient, causing many high-risk individuals to miss the optimal window for intervention. Second, the test requires patients to cooperate properly with the procedure of “deep inhalation followed by forced exhalation.” Older adults, individuals with obesity, patients with cognitive impairment, and those with severe cardiopulmonary comorbidities often have poor cooperation, which increases the risk of misclassification. In addition, clinical symptom questionnaires are highly subjective and have low sensitivity; symptom-based assessment lacks objective criteria and therefore has limited predictive performance. PRISm may be predicted using imaging-derived metrics, with Computed Tomography (CT) providing quantitative information on lung volume, pulmonary density, airway features, and vascular anatomy, and Magnetic Resonance Imaging (MRI) offering direct assessment of ventilation capacity and pulmonary perfusion (9). Nevertheless, CT involves radiation exposure, and MRI is costly, making these modalities unsuitable for short-term repeated screening and large-scale health examinations. Therefore, objective, inexpensive, and easily accessible biomarkers are urgently needed for PRISm screening and prediction.

In view of these screening challenges, routine peripheral blood testing represents an ideal option because it is convenient, low-cost, and provides objective quantitative results. Testing can be completed in primary healthcare settings using only 2–5 mL of venous blood. Complete blood count combined with biochemical parameters is inexpensive, unaffected by subjective factors, and suitable for large-scale screening. The C-reactive protein-albumin-lymphocyte index (CALLY index), a composite hematologic marker derived from CRP, albumin, and lymphocyte count, was initially developed by Iida H et al. and offers a multidimensional assessment encompassing systemic inflammation, nutritional reserve, and immunological competence (10). This index has been widely applied in prognostic assessment across various cancers and is regarded as a clinically feasible biomarker (11–15). In respiratory diseases, the CALLY index has been shown to be an important predictor of asthma-chronic obstructive pulmonary disease overlap (16). A high CALLY index reflects a low-inflammatory, well-nourished, and immunocompetent status, whereas a low value indicates systemic imbalance. This profile is highly consistent with the pathogenesis of PRISm, in which chronic inflammation, malnutrition, and impaired immune function jointly promote the progression of lung function toward PRISm (17–19). The present investigation, premised on the foregoing rationale, hypothesized that a higher CALLY index is independently associated with lower odds of PRISm, and uniquely assessed its added predictive utility beyond a traditional baseline model, aspiring to introduce an efficient and novel biomarker applicable to PRISm case identification and individualized risk categorization.

## 2 Materials and methods

### 2.1 Study population

**FIGURE 1:**
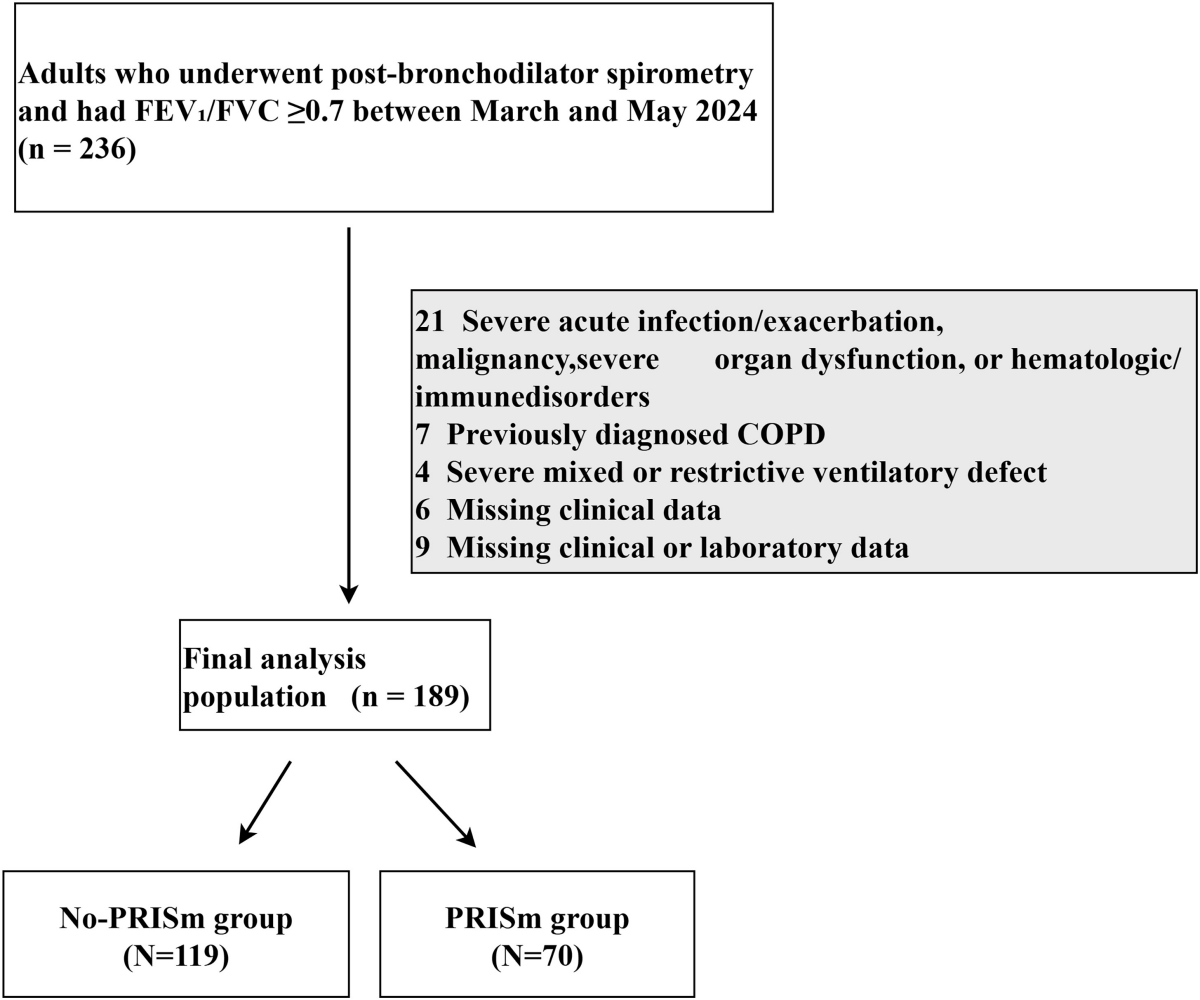
Flowchart of participant selection.

This study was designed as a retrospective observational study. Consecutive participants who visited The Central hospital of Enshi Tujia and Miao Autonomous Prefecture and underwent spirometry between March 2024 and May 2024 were enrolled. The inclusion criteria were as follows: (1) age ≥ 18 years; (2) post-bronchodilator FEV1/FVC ≥ 0.7; and (3) availability of a complete spirometry report and clinical data. The exclusion criteria were as follows: (1) severe infection or respiratory disease in the acute exacerbation phase; (2) concomitant malignancy, severe hepatic or renal dysfunction, or hematologic or immune disorders; (3) previously diagnosed chronic obstructive pulmonary disease or restrictive ventilatory defects. Restrictive lung disease was excluded based on a comprehensive evaluation of full pulmonary function testing (including total lung capacity [TLC]), chest imaging findings, and clinical history; and (4) missing clinical or laboratory data. Ultimately, 189 eligible participants were included in the statistical analysis (Figure 1). The study protocol was approved by the Ethics Committee of Enshi Tujia and Miao Autonomous Prefecture Central Hospital (approval No. 2024-063-01). Given the retrospective nature of the study, the requirement for informed consent was waived by the ethics committee.

### 2.2 Data collection and variable definitions

Baseline clinical information was extracted from the hospital’s computerized patient record platform. Age, gender, body mass index (BMI), and smoking history were documented as the core sociodemographic and lifestyle covariates in this study. Hypertension, diabetes mellitus, and coronary heart disease were recorded as concurrent pathological conditions, each verified against historically documented clinical diagnoses. Venous blood collected under fasting conditions served as the biological matrix for quantifying a series of hematological parameters, including leukocyte count, neutrophil count, monocyte count, eosinophil count, erythrocyte count, hemoglobin, platelet count, and red blood cell distribution width-coefficient of variation (RDW-CV). Constituting the main exposure of analytical focus, the CALLY index was operationalized via the following arithmetic expression: albumin (g/L) × lymphocyte count ( × 10^9^/L)/[CRP (mg/L) × 10] (12). Consistent with the widely accepted clinical consensus definition—post-bronchodilator forced expiratory volume in 1 s/forced vital capacity ≥ 0.70 with FEV1 < 80% predicted (1)—PRISm was designated as the endpoint variable of the current analysis. Post-bronchodilator pulmonary function parameters, including percent predicted FVC (FVC% predicted), percent predicted FEV1 (FEV1% predicted), and FEV1/FVC ratio, were also collected to characterize the study population. Based on outcome status, all participants were stratified into two cohorts: the PRISm group and the non-PRISm group.

### 2.3 Statistical analyses

Continuous variables for baseline characteristics were expressed as mean ± standard deviation (mean ± SD) if they followed a normal distribution, and between-group comparisons were performed using the independent-samples Student’s *t*-test. Categorical variables were presented as frequencies and percentages (*n*, %), and between-group comparisons were conducted using the chi-square test. To evaluate the independent association between the CALLY index and the odds of PRISm, three sequentially adjusted multivariable Logistic regression models were constructed. All 189 participants had complete data on all included variables, and no imputation was required. The unadjusted model included no covariates. Adjusted model I was adjusted for gender, age, smoking history, and BMI. Adjusted model II was further adjusted for hypertension, diabetes mellitus, coronary heart disease, red blood cell count, hemoglobin, platelet count, and RDW-CV on the basis of adjusted model I. Covariates were selected based on clinical relevance, including demographic characteristics, comorbidities, and laboratory parameters that are established or potential risk factors for impaired lung function. Multicollinearity was assessed using variance inflation factors (VIF) with ah threshold of < 5. The CALLY index was entered into the models as a continuous variable, and as a dichotomous variable, categorized according to the median value (0.906) in the overall study population. The results were expressed as odds ratios (ORs) and 95% confidence intervals (95% CIs).

In addition, Logistic regression-based restricted cubic spline (RCS) models were used to explore the dose-response relationship between the continuous CALLY index and the risk of developing PRISm. To assess the robustness of the findings, subgroup analyses were further performed according to clinical characteristics, including age, gender, smoking history, and comorbidities, and forest plots were generated. P values for interaction were calculated. Finally, to evaluate the incremental predictive value of the CALLY index, the area under the receiver operating characteristic (ROC) curve (AUC) was compared between the baseline model (Model 2: age + gender + smoking history + BMI) and the combined model incorporating the CALLY index (Model 1). Differences between the two models were assessed using the DeLong test. Decision curve analysis (DCA) was further performed to evaluate the net clinical benefit of the combined model. All statistical analyses were conducted using R software (version 4.3.0, R Foundation) and EmpowerStats (X&Y Solutions, Inc.). A two-sided *P* < 0.05 was considered statistically significant.

## 3 Results

### 3.1 Baseline characteristics of the study population

Table 1 presents the baseline clinical characteristics of the 189 participants included in this study. Of these, 119 participants were assigned to the non-PRISm group, accounting for 63.0%, and 70 participants were assigned to the PRISm group, accounting for 37.0%. No statistically significant differences were observed between the two groups in age (61.0 ± 13.1 vs. 60.8 ± 12.1 years, *P* = 0.933), BMI, gender distribution, or the prevalence of previous cardiovascular and metabolic comorbidities, including hypertension, diabetes mellitus, and coronary heart disease. Regarding laboratory parameters, the PRISm group showed significant differences in white blood cell count (*P* = 0.002), neutrophil count (*P* < 0.001), C-Reactive Protein (*P* < 0.001) and eosinophil count (*P* = 0.025). In terms of pulmonary function, the PRISm group exhibited significantly lower post-bronchodilator FVC% (67.7 ± 10.1 vs. 103.1 ± 15.2, *P* < 0.001) and FEV1% (68.7 ± 9.2 vs. 101.5 ± 14.9, *P* < 0.001), while the FEV1/FVC ratio remained above 0.70 in both groups (82.2 ± 6.2 vs. 79.7 ± 5.3, *P* = 0.023). Notably, the CALLY index was significantly lower in the PRISm group than in the non-PRISm group (1.4 ± 2.5 vs. 4.7 ± 8.1, *P* < 0.001), preliminarily suggesting that a lower CALLY index may be closely associated with the presence of PRISm.

**TABLE 1:** Baseline characteristics of the study population according to PRISm status.

Characteristic	No-PRISm (*N* = 119)	PRISm (*N* = 70)	*P*-value
Age	61.0 ± 13.1	60.8 ± 12.1	0.933
BMI	23.9 ± 3.4	23.0 ± 4.1	0.099
White blood cell count	6.2 ± 2.6	7.7 ± 3.5	0.002
Neutrophil count	4.1 ± 2.3	5.7 ± 3.3	< 0.001
Monocyte count	0.4 ± 0.2	0.5 ± 0.2	0.143
Eosinophil count	0.2 ± 0.2	0.1 ± 0.1	0.025
Red blood cell count	4.1 ± 0.6	4.0 ± 0.6	0.517
Hemoglobin	123.2 ± 18.3	120.5 ± 19.1	0.341
C-reactive protein	17.4 ± 36.8	48.9 ± 71.2	< 0.001
Platelet count	216.7 ± 85.2	230.4 ± 84.7	0.288
CALLY	4.7 ± 8.1	1.4 ± 2.5	< 0.001
RDW-CV	13.5 ± 1.5	13.5 ± 1.8	0.966
FVC (% predicted)	103.1 ± 15.2	67.7 ± 10.1	< 0.001
FEV1 (% predicted)	101.5 ± 14.9	68.7 ± 9.2	< 0.001
FEV1/FVC (%)	79.7 ± 5.3	82.2 ± 6.2	0.023
**Gender**			0.081
Female	58 (48.7%)	25 (35.7%)	
Male	61 (51.3%)	45 (64.3%)	
**Smoking history**			0.062
No	76 (63.9%)	35 (50.0%)	
Have	43 (36.1%)	35 (50.0%)	
**Hypertension**			0.269
No	95 (79.8%)	51 (72.9%)	
Have	24 (20.2%)	19 (27.1%)	
**Diabetes mellitus**			0.096
No	107 (89.9%)	57 (81.4%)	
Have	12 (10.1%)	13 (18.6%)	
**Coronary heart disease**			0.281
No	117 (98.3%)	67 (95.7%)	
Have	2 (1.7%)	3 (4.3%)	

PRISm, preserved ratio impaired spirometry; CALLY, C-reactive protein-albumin-lymphocyte index; BMI, body mass index; WBC, white blood cell count; RDW-CV, red cell distribution width–coefficient of variation; FVC, forced vital capacity; FEV1, forced expiratory volume in 1 s.

### 3.2 Multivariable association analysis between the CALLY index and the odds of PRISm

Multivariable Logistic regression models were used to evaluate the independent predictive value of the CALLY index for PRISm (Table 2). When the CALLY index was analyzed as a continuous variable, in the most stringent model after full adjustment for 11 confounding factors, including demographic characteristics, comorbidities, and peripheral blood parameters (adjusted model II), each one-unit increase in the CALLY index was associated with 16% lower odds of PRISm (OR = 0.84, 95% CI: 0.74–0.95, *P* = 0.007). To further clarify its clinical relevance, the CALLY index was additionally entered into the model as a dichotomous variable according to the median value (0.906). In the dichotomous analysis, the odds of PRISm were significantly lower in the high-CALLY group in both the unadjusted model and the core-variable adjusted model (adjusted model I). Even in the fully adjusted model (adjusted model II), participants with a high CALLY index had only 27% of the odds of PRISm observed in those with a low CALLY index (OR = 0.27, 95% CI: 0.13–0.57, *P* < 0.001). These findings provide strong evidence that a high CALLY index is independently associated with lower odds of PRISm.

**TABLE 2:** Association between the CALLY index and PRISm using logistic regression models with progressive adjustment.

Exposure	Non-adjusted	Adjust I	Adjust II
	β (95%CI) *P*-value / OR (95%CI) *P*-value		
CALLY median value (0.906)			
Low	1	1	1
High	0.26 (0.14, 0.49) < 0.001	0.27 (0.14, 0.53) < 0.001	0.27 (0.13, 0.57) < 0.001
CALLY	0.83 (0.73, 0.94) 0.003	0.84 (0.74, 0.95) 0.005	0.84 (0.74, 0.95) 0.007

Odds ratios (ORs) and 95% confidence intervals (CIs) were estimated using logistic regression. The non-adjusted model included no covariates. Adjust I was adjusted for gender, age, smoking history, and BMI. Adjust II was further adjusted for gender, age, smoking history, BMI, hypertension, diabetes mellitus, coronary heart disease, red blood cell count, hemoglobin, platelet count, and RDW-CV. OR, Odds ratio; CI, confidence interval; CALLY, C-reactive protein-albumin-lymphocyte index; PRISm, preserved ratio impaired spirometry; BMI, body mass index; RDW-CV, red cell distribution width–coefficient of variation.

### 3.3 Dose-response relationship and sensitivity subgroup analyses

**FIGURE 2:**
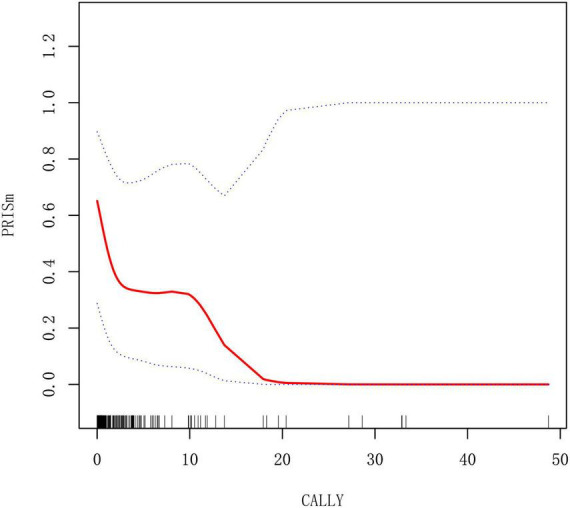
Dose–response relationship between the CALLY index and the odds of PRISm assessed by restricted cubic spline. CALLY, C-reactive protein-albumin-lymphocyte index; PRISm, preserved ratio impaired spirometry.

**FIGURE 3:**
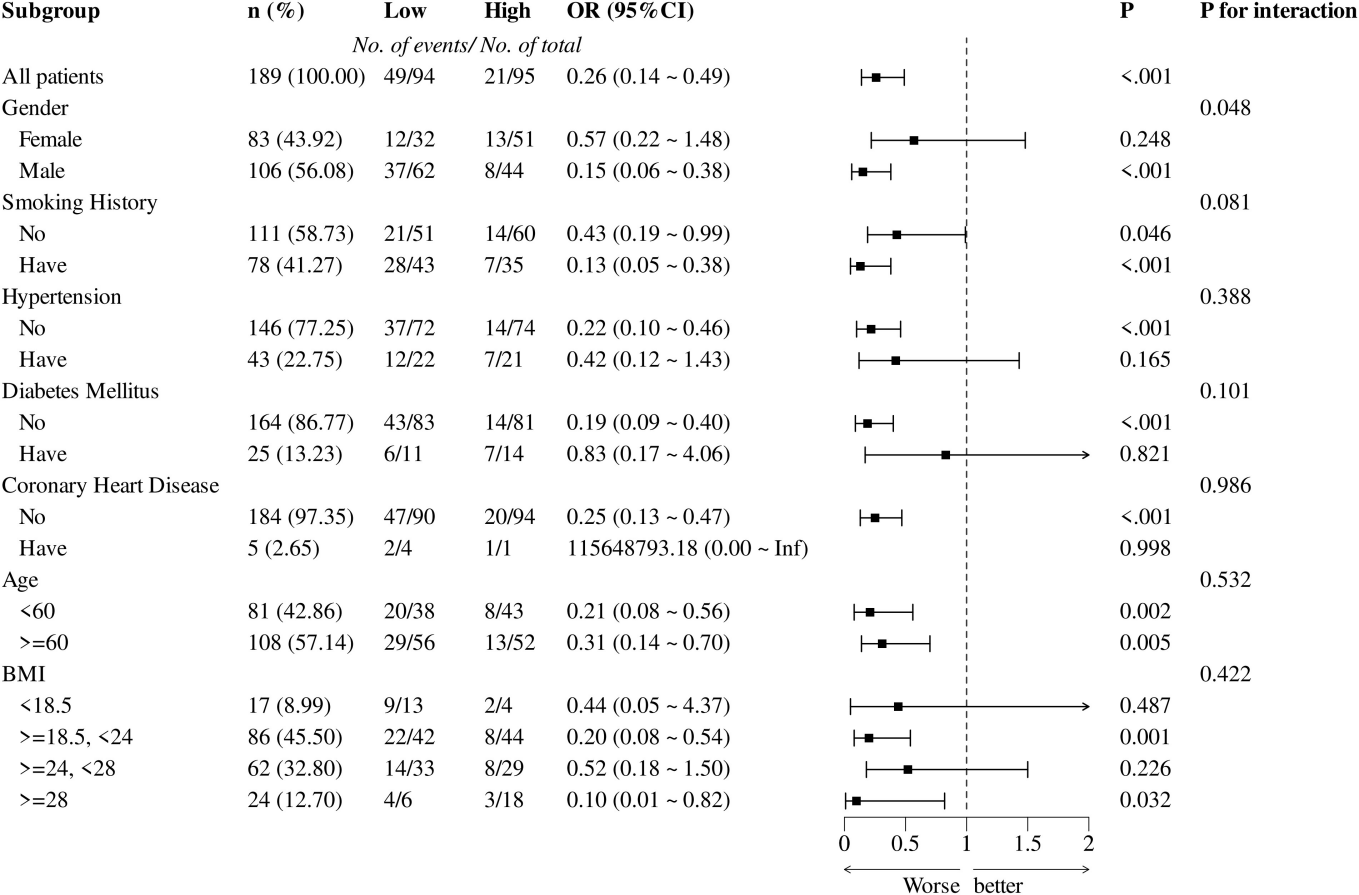
Subgroup analysis for the association between CALLY index (dichotomized) and PRISm across different clinical strata. CALLY,C-reactive protein-albumin-lymphocyte index; PRISm, preserved ratio impaired spirometry.

To visualize the dynamic association between the CALLY index and the odds of PRISm, restricted cubic spline (RCS) curves were plotted (Figure 2). The curve clearly showed that, as the CALLY index gradually increased, the odds of PRISm among participants exhibited a sustained and significant downward trend, further supporting the inverse association identified in the regression models. Subsequently, to assess the robustness of this inverse association in specific populations, subgroup analyses were performed across multiple strata, including age, gender, smoking history, BMI, hypertension, and diabetes mellitus (Figure 3). The forest plot showed that the inverse association between a high CALLY index and PRISm remained consistent across most subgroups. Notably, this inverse association was particularly pronounced among men (OR = 0.15, *P* < 0.001), smokers (OR = 0.13, *P* < 0.001), and individuals without diabetes mellitus (OR = 0.19, *P* < 0.001). No significant interaction effects were observed across subgroups, with most *P* values for interaction > 0.05, indicating that the CALLY index has strong clinical generalizability as an independently associated factor.

### 3.4 Incremental clinical predictive value of the CALLY index for PRISm

**FIGURE 4:**
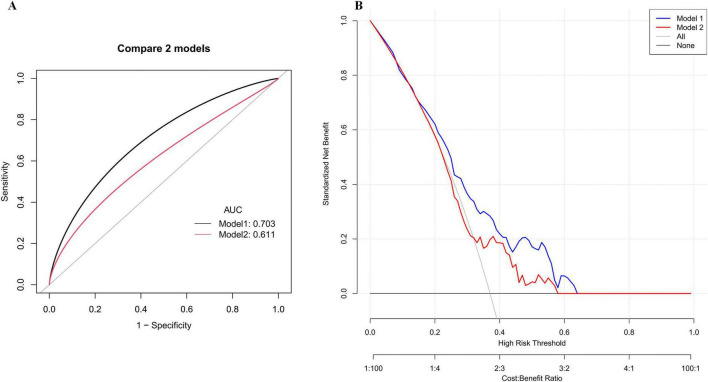
Incremental predictive value of the CALLY index beyond the baseline clinical model. **(A)** Comparison of receiver operating characteristic (ROC) curves between Model?1 (baseline model+CALLY index) and Model2 (baseline model including age, gender, smoking history, and BMI). **(B)** The decision curve analysis showing the standardized net benefit of Model1 versus Model2 across a wide range of threshold probabilities. CALLY, C-reactive protein-albumin-lymphocyte index; BMI, body mass index.

Finally, we investigated the incremental value of incorporating the CALLY index into a routine clinical assessment model (Figure 4). The baseline model containing only basic clinical characteristics, including age, gender, smoking history, and BMI (Model 2), yielded an AUC of 0.611 (95% CI: 0.527–0.690) for predicting PRISm. After the CALLY index was added to Model 2 to construct a new model (Model 1), the AUC increased markedly to 0.703 (95% CI: 0.628–0.774). The DeLong test demonstrated that this improvement in predictive performance was highly statistically significant (*P* = 0.003). In addition, decision curve analysis (DCA) further confirmed that, across a very broad range of risk threshold probabilities, Model 1 incorporating the CALLY index provided a higher standardized net clinical benefit for patients than the baseline model.

## 4 Discussion

A total of 189 participants were included in this study. Multivariable Logistic regression analysis showed that the CALLY index was significantly inversely associated with the prevalence of PRISm. To further assess the precision and robustness of this association, additional subgroup analyses were performed. These analyses indicated that the inverse association remained stable across subgroups stratified by age, gender, smoking history, and comorbidities. Moreover, after the CALLY index was added to the model incorporating baseline clinical characteristics, the area under the receiver operating characteristic curve (AUC) increased significantly from 0.611 to 0.703 (*P* = 0.003). The inverse association between the CALLY index and the prevalence of PRISm became more pronounced, and DCA further confirmed its greater net clinical benefit.

The CALLY index, rather than functioning as an individual laboratory parameter, but a composite index calculated from three routine blood test parameters. It is designed to provide an integrated assessment of the body’s internal milieu across three dimensions: inflammation, nutrition, and immunity. Specifically, a higher albumin level reflects better nutritional status and synthetic function; a higher lymphocyte count indicates more active adaptive immune function; and a lower CRP level represents a lower degree of systemic inflammation (20, 21). Albumin contains multiple binding sites within its molecular structure that can chelate pro-oxidant ions such as free iron and copper. It is also the principal thiol-containing protein in plasma, exerting direct antioxidant and anti-inflammatory effects. Adequate serum albumin levels provide essential substrates for the regeneration and repair of alveolar epithelial cells and capillary endothelial cells, thereby helping to maintain the structural integrity of the alveolar-capillary barrier (22–24). As an open organ, the lung is continuously exposed to pathogens and environmental irritants. Sufficient lymphocyte reserves, particularly effector T cells and B cells, are therefore essential for effective immune surveillance. When immune homeostasis is maintained, as reflected by a high CALLY index, Th2 cell activity is appropriately regulated, which may help suppress eosinophilic infiltration of the airways and excessive mucus secretion (25). Elevated CRP levels can further activate the complement system and recruit additional inflammatory cells, such as neutrophils and macrophages, thereby perpetuating a vicious inflammatory cycle. A high CALLY index suggests that this cascade is substantially attenuated (26, 27).

PRISm is, in essence, not merely a localized organ disorder, but rather a “systemic syndrome” accompanied by multisystem involvement, particularly cardiometabolic abnormalities (28, 29). Precisely because of the systemic nature of PRISm, a composite systemic indicator such as the CALLY index may be better suited to capture the deeper pathophysiological abnormalities underlying PRISm than a single pulmonary marker or an isolated blood parameter, such as white blood cell count or hemoglobin alone. The CALLY index was initially widely used for prognostic assessment in various solid tumors, including hepatocellular carcinoma and gastrointestinal tumors (30–32). In recent years, its application has extended to cardiovascular diseases, such as heart failure and coronary heart disease, as well as critical care medicine, demonstrating its considerable potential as a marker of systemic homeostatic imbalance (33, 34). Several studies have shown that the CALLY index is associated with better preservation of lung function. In chronic obstructive pulmonary disease and asthma-COPD overlap, an increased CALLY index has been significantly associated with a lower odds of disease, with each one-log-unit increase corresponding to lower odds of ACO of up to 43% (16). A high CALLY index indicates an ideal homeostatic state characterized by low inflammation, favorable nutritional status, and strong immune competence. These three mechanisms act synergistically, enabling lung tissue to better withstand injurious stimuli, repair damage, and thereby maintain better pulmonary function. The present study further expands the application scope of the CALLY index.

Our study confirms that, even in the presence of established risk factors such as smoking, age, and BMI, the CALLY index continues to provide an additional predictive value. The subgroup analysis shown in Figure 3 further supports this finding, with the inverse association being particularly pronounced among smokers and men. Additionally, the accurate diagnosis and classification of PRISm may be influenced by the choice of spirometric reference equations across different populations, as recently highlighted by Loeloe et al., who developed machine learning-based spirometry reference values tailored to the Iranian population (35). These findings underscore the importance of population-specific reference standards in PRISm research, which should be considered in future multi-ethnic validation studies. Moreover, as shown in Figure 4, the conventional prediction model (Model 2), which included only static demographic characteristics, failed to capture the patient’s current dynamic physiological status, resulting in a relatively low AUC. After the CALLY index was incorporated (Model 1), this limitation was substantially addressed. These findings provide an ideal, low-cost, and efficient approach for screening populations at high risk of PRISm in primary healthcare institutions.

As a retrospective/cross-sectional analytical framework was adopted, the strong correlation identified notwithstanding, causality between the CALLY index and PRISm cannot be conclusively inferred. In addition, the sample size was relatively limited (*N* = 189), with all participant information sourced from one institutional site. The fully adjusted logistic regression model included 11 covariates with 70 PRISm events (events-per-variable ratio ≈ 6.4), which is a recognized limitation. Although the primary findings remained consistent across models with varying degrees of adjustment (from 4 to 11 covariates), the possibility of model overfitting cannot be entirely excluded given the limited number of outcome events. Future studies with larger sample sizes are warranted to validate these findings. Although the internal robustness of our findings was confirmed through rigorous statistical methods, including nonlinear testing and subgroup analyses, an external validation cohort remains lacking. Internal validation using bootstrap resampling was not performed, which should be considered when interpreting the stability of the regression estimates. Although the exclusion of restrictive lung disease was based on a comprehensive evaluation of full pulmonary function testing (including TLC), chest imaging, and clinical assessment, subclinical restrictive defects cannot be completely ruled out with absolute certainty. Several potential factors that may affect pulmonary function, including exposure to ambient air pollution, occupational dust exposure, a history of childhood respiratory tract infections, and detailed recent medication use, could not be fully included in the model adjustments because of the limitations inherent to the retrospective data.

Irrespective of the aforementioned constraints, the present analysis establishes a high CALLY index as independently associated with lower odds of PRISm. As an easily obtainable, cost-effective, and efficient clinical tool, the CALLY index may assist physicians in the early identification and protection of individuals with potential respiratory health impairment during routine health examinations and primary care practice.

## 5 Conclusion

From retrospective data involving 189 participants, it was preliminarily noted that an elevated CALLY index was independently and inversely associated with the odds of PRISm, with the corresponding inverse trend exhibiting general robustness across stratifications by age, sex, smoking history, and comorbidities. After the CALLY index was added to the baseline clinical model, both predictive discrimination and net clinical benefit improved to some extent. Given its simplicity and cost-effectiveness, the CALLY index, a composite biomarker, may furnish a useful reference for the initial identification of population subsets bearing heightened PRISm vulnerability; however, its precise clinical translational value remains to be further clarified.

## Reporting guidelines

This study is reported following the RECORD (REporting of studies Conducted using Observational Routinely-collected health Data) guidelines (36), to ensure comprehensive and transparent reporting of this observational study using routinely collected health data.

## Data Availability

The raw data supporting the conclusions of this article will be made available by the authors, without undue reservation.
